# PPAR*β*/*δ*-Interfering Peptide Enhanced Mesenchymal Stromal Cell Immunoregulatory Properties

**DOI:** 10.1155/2022/5494749

**Published:** 2022-12-13

**Authors:** Gautier Tejedor, Prisca Boisguerin, Éric Vivès, Christian Jorgensen, Jérôme Guicheux, Claire Vinatier, Claire Gondeau, Farida Djouad

**Affiliations:** ^1^IRMB, University of Montpellier, INSERM, Montpellier, France; ^2^MedXCell Science SAS, Montpellier, France; ^3^PhyMedExp, University of Montpellier, INSERM, CNRS, Montpellier, France; ^4^CHU Montpellier, Montpellier F-34295, France; ^5^Oniris, CHU Nantes, INSERM, Regenerative Medicine and Skeleton, RMeS, Nantes Université, UMR 1229, F-44000 Nantes, France

## Abstract

**Background:**

Mesenchymal stem/stromal cells (MSCs) have been widely used for their therapeutic properties in many clinical applications including osteoarthritis. Despite promising preclinical results showing the ability of MSC to reduce the clinical severity of osteoarthritis (OA) in experimental animal models, the benefits of intra-articular injection of MSC in OA patients are limited to the short term. In this regard, it is anticipated that improving the properties of MSC may collectively enhance their long-term beneficial effects on OA.

**Methods and Results:**

Recently, we have shown that PPAR*β*/*δ* inhibition using a commercially available antagonist in murine MSC increases their immunoregulatory potential *in vitro* as well as their therapeutic potential in an experimental murine arthritis model. Here, we relied on an innovative strategy to inhibit PPAR*β*/*δ*:NF-*κ*B TF65 subunit interaction in human MSC by designing and synthesizing an interfering peptide, referred to PP11. Through RT-qPCR experiments, we evidenced that the newly synthesized PP11 peptide reduced the expression level of *PDK4*, a PPAR*β*/*δ* target gene, but did not modify the expression levels of *ACOX1* and *CPT1A*, PPAR*α* target genes, and *FABP4*, a PPAR*γ* target gene compared with untreated human MSC. Moreover, we showed that human MSCs pretreated with PP11 exhibit a significantly higher capacity to inhibit the proliferation of activated PBMC and to decrease the frequency of M1-like macrophages.

**Conclusions:**

We designed and synthesized an interfering peptide that potently and specifically blocks PPAR*β*/*δ* activity with concomitant enhancement of MSC immunoregulatory properties.

## 1. Introduction

Strategies to improve mesenchymal stem/stromal cell (MSC) therapeutic properties for osteoarthritis (OA) treatment by enhancing in particular, immunoregulatory, and cytoprotective properties are a matter of intense research. These strategies consist, in part, of genetic or pharmacological preconditioning of MSC to further enhance the expression of one candidate gene or to activate a specific pathway.

Recently, we demonstrated that PPAR*β*/*δ*, a lipid ligand-inducible transcription factor described for its metabolic functions, plays a key role in MSC derived from murine bone marrow (mMSC) through its capacity to regulate their NO production [1]. This suppressive role of PPAR*β*/*δ* was mediated through the regulation of NF-*κ*B activity, a major player in MSC immunosuppressive properties [1, 2]. Murine MSC from PPAR*β*/*δ* knockout mice exhibited increased immunosuppressive potential which was interestingly correlated with an increased NF-*κ*B activity both at a steady state and upon activation with proinflammatory cytokines [1]. Thus, PPAR*β*/*δ* inhibition in MSC increased their immunoregulatory potential *in vitro*, on activated splenocytes or T-cell subsets, and their therapeutic potential in an experimental murine arthritis model [3]. Indeed, primary function of PPARs during the inflammatory response is to promote NF-*κ*B inactivation. Possible mechanisms of inactivation could include inhibition through direct binding and ubiquitination leading to proteolytic degradation of NF-*κ*B TF65 as well as indirect effects on NF-*κ*B [4].

Additionally, whether PPAR*β*/*δ* modulation using commercially available PPAR*β*/*δ* antagonists in human MSC could affect their immunoregulatory potential has never been investigated. Moreover, the nonspecificity of the current available PPAR*β*/*δ* antagonists that can modulate the activity of other members of the PPAR family is well documented [5], as well as the fact that the MSC culture media serum contains PPAR*β*/*δ* ligands [6]. Indeed, PPAR ligands are present in the serum of the cell culture media and can be produced by the cells themselves [6]. Thus, according to the cell type used in studies (for instance human versus mouse MSC), different concentrations of PPAR ligands can accumulate within the culture media.

In addition to the influence of ligands on the conformation of PPAR*β*/*δ*, the molecular environment of the nuclear receptor (presence or absence of coactivators/corepressors) might also influence its activity [7, 8]. Moreover, at this time, despite the role of PPAR*β*/*δ* in many cellular mechanisms, the synthesis of PPAR*β*/*δ* antagonists or reverse agonists are limited to three compounds (antagonists: GSK3787, SR13904 or reverse agonist: GSK0660), and no synthetic ligand of PPAR*β*/*δ* has received marketing authorization.

PPAR*β*/*δ* can interact directly with other transcription factors, including NF-*κ*B, by blocking their nuclear translocation and/or their binding to the DNA. Given that the NF-*κ*B signaling pathway is required for the initial priming of immunosuppressive function in human MSC [2], in the present study, we relied on an innovative strategy to inhibit PPAR*β*/*δ*:NF-*κ*B TF65 subunit interaction by designing an interfering peptide. Then, we studied the specificity of this peptide mimicking the NF-*κ*B TF65 subunit in adipose tissue-derived MSC (AD-MSC) by evaluating (i) the expression levels of PPAR family member target genes, (ii) their immunoregulatory properties on activated human peripheral blood mononuclear cells (PBMC) and the expression level of MSC immunoregulatory mediators, and (iii) their immunoregulatory properties on macrophages.

## 2. Materials and Methods

### 2.1. Peptide Array Synthesis

All peptide arrays were synthesized with a ResPep synthesizer (Intavis AG) using the standard SPOT synthesis as described in detail previously [9]. Sequence files were generated with the help of the software LISA 1.78 (in-house software). 0.4 M Fmoc-protected Opfp-esters (Bachem) in NMP (Fluka) were coupled on a beta-alanine-modified cellulose membrane (Whatman-50) to generate the peptide arrays [9]. The following side-chain protection groups were used: tert-butyl ester (OtBu) for E and D; tert-butyl ether (tBu) for S, T, and Y; triphenylmethyl (Trt) for C, N, Q, and H, 2,2,4,6,7-pentamethyldihydrobenzofuran-5-sulfonyl (Pbf) for R; and tert-butoxycarbonyl (Boc) for K and W. Fmoc deprotection was performed using a 20% piperidine solution (in DMF) for 15 min. Side-chain protecting group cleavage was performed using two trifluoroacetic acid (TFA, Sigma-Aldrich) solutions (90% TFA, 2% H_2_0, 3% triisobutylsilane (TIBS, Sigma-Aldrich), and 10% dichloromethane (DCM, Sigma-Aldrich); and 60 TFA, 2% H_2_0, 3% TIBS, and 35% DCM) [10].

The following libraries were synthesized twice: PepScan (15-mer overlapping peptides with a shift of 3 amino acids); length analysis (18-mer peptide shorted from N- and C-terminus); substitutional analysis (each amino acid of the PP11 peptide replaced by the 20 L-amino acids).

### 2.2. Peptide Array Binding Studies

The cellulose membrane-bound peptides were prewashed once with ethanol (1 × 10 min), with tris-buffered saline (TBS), pH 8.0, (3 × 10 min), and then blocked for 3 h with blocking buffer (Sigma-Aldrich) in TBS pH 8.0, containing 5% sucrose (Sigma-Aldrich). The membranes were incubated with human recombinant PPAR*β*/*δ* protein (Leu-260-Glu426; PPARD-15H human, Interchim) having an N-terminal His-tag for the detection (5 *μ*g/ml in blocking buffer overnight at 4°C). The next day, the membrane was washed with TBS (3 × 10 min) and incubated with a primary anti-His antibody (H1029, Sigma-Aldrich; 1 : 1,000 in blocking buffer) for 2.5 h at room temperature. After TBS washes (3 × 10 min) to remove the primary antibody, the membrane was incubated with a peroxidase-labeled anti-mouse-HRP (#7076S, Cell Signaling; 1 : 2,000 in blocking buffer) for 1.5 h at room temperature. To remove antibody excess, the membrane was washed with TBS (3 × 10 min) and then incubated with Pierce ECL Plus Western Blotting Substrate (Thermo Fisher). Images were acquired with an Imager 600 (Amersham), and spot intensities were measured using the “protein array analyzer” macro of ImageJ. An arbitrary threshold was set for each membrane at 100,000 to determine the “binders” (see Supplementary materials, Tables S1, S2, and S3).

### 2.3. Peptide Synthesis

PP11 peptide (Ac-GRKKRRQRRR-RISLVTKDPPHR-CONH_2_, 22-mer) was synthesized on a Liberty Blue™ Microwave Peptide Synthesizer (CEM Corporation) with an additional module of Discover™ (CEM Corporation) combining microwave energy at 2450 MHz to the fluorenylmethoxycarbonyl (Fmoc)/tert-butyl (tBu) strategy (SynBio3 platform). Peptide identity and purity (>95%) were checked by LC-MS (Waters).

### 2.4. Human Adipose-Derived Stromal Cell Isolation and Expansion in Hollow-Fiber Bioreactor

Human adipose-derived stromal cells (hAD-MSCs) were isolated from abdominal adipose tissue obtained from 6 healthy donors (*N* = 6) receiving aesthetic surgery and were expanded *in vitro*. In brief, lipoaspirate from each donor was washed twice with a buffered-saline solution to remove residual blood. The adipose tissue was digested by incubation with collagenase NB4 (Nordmark). The collagenase was neutralized with cell culture media (Minimum Essential Medium, *α*-MEM, Gibco) containing 5% human platelet lysate (hPL Stemulate, Sexton Biotechnologies), and the suspension was filtered, centrifuged, and resuspended in complete media. The number of mononucleated cells (MNCs) in the isolated stromal vascular fraction (SVF) and cell viability was determined using an automated cell counter (NucleoCounter® NC-200™, ChemoMetec). SVF was then resuspended in the cell culture media at the desired concentration and loaded into the intracapillary space of the hollow fibers of the Quantum® Cell Expansion System (Terumo BCT). Expansion of MSC isolated from adipose tissue or bone marrow has been previously described by others ([11, 12], respectively). Briefly, 24 h before cell loading, the bioreactor was coated with fibronectin from human plasma (Corning, NY) to support attachment and expansion of MSC. Over the course of cell expansion (7-8 days), the feed rate was adjusted according to the manufacturer's instructions. After trypsinization and washing steps with a buffered-saline solution, hAD-MSC (passage P0) were cryopreserved in a cryopreservation media (Cryostor® CS10, BioLife Solutions). hAD-MSCs used in this study were obtained after a second expansion step in the Quantum® system (passage P1, 6 days of cultivation) and cryopreserved in Cryostor® CS10 (BioLife Solutions). Of note, the telomere-associated-protein Rap1 (Trf2IP) was not or weakly expressed in hAD-MSC from the 6 donors used in that study.

### 2.5. Human Adipose-Derived Stromal Cell Pretreatment

Just after thawing, hAD-MSCs were plated at a density of 4,500 cells/cm^2^ in 5% hPL-supplemented *α*-MEM containing 1% of penicillin-streptomycin (Gibco) for 48 hours. Then, hAD-MSCs were treated with either PPAR*β*/*δ* antagonist (GSK3787) (Sigma) or PP11 peptide at a concentration of 0.1 *μ*M for 24 h.

### 2.6. Quantitative Real-Time Polymerase Chain Reaction (qRT-PCR)

RNA extraction was performed using RNeasy Mini kit (Qiagen) according to the manufacturer's instructions. A total amount of 500 ng of RNA was used to retrotranscribe in cDNA with the SensiFAST cDNA Synthesis kit (Meridian). Real-time rt-qPCR was done following the protocol of SYBR SensiFAST No-ROX Kit (Meridian) and a LightCycler 480 Detection system (Roche Applied Science). The primer sequences were designed with the Primer3 software. The normalization was performed with ribosomal protein S9 (RPS9) as a housekeeping gene and compared to conditions with hAD-MSC without treatment. The results are expressed as relative mRNA levels of gene expression and obtained thanks to the 2^−ΔΔCt^ formulae.

### 2.7. Immunosuppression Assay

Human peripheral blood mononuclear cells (PBMCs) from 3 different healthy donors (Etablissement Français du Sang) were isolated using the Ficoll procedure and frozen in DMSO supplemented with 10% fetal calf serum. Frozen PBMCs were thawed, counted, and labeled with CellTrace Violet (CTV, Thermo Fisher) to assess their proliferation rate. PBMCs were activated with phytohemagglutinin (PHA; 5 mg/ml) (Thermo Fisher Scientific). PHA-activated PBMCs were then cultured in the presence or absence of hAD-MSC pretreated or not for 24 h with the antagonist (GSK3787, 0.1 *μ*M) or with the interfering PP11 peptide (0.1 *μ*M) at a cell ratio of 1 : 10 (hAD-MSC:PBMC) in a mixed lymphocyte reaction (MLR) medium, containing 10% FCS (Biosera, France), 1% penicillin-streptomycin (Gibco), 1% sodium pyruvate (Thermo Fisher Scientific), 1% nonessential amino acids (Thermo Fisher Scientific), and 1% glutamine (Thermo Fisher Scientific) in Iscove's Modified Dulbecco's Media (IMDM—Thermo Fisher Scientific). After 96 hours of coculture, PHA-activated PBMC proliferation was quantified by flow cytometry using the BD FACSCanto (MRI platform). To determine the percentage of inhibition of PBMC proliferation by hAD-MSC, PHA-activated PBMC was considered as 100% of proliferation; hAD-MSC decrease the percentage of PHA-activated PBMC proliferation; the difference between both conditions corresponds to the percentage of inhibition.

### 2.8. Macrophage Polarization Assay

Human monocytes were isolated from leukocyte concentrate (buffy-coat) from healthy donors. Human PBMCs were obtained by the Ficoll-Paque density gradient centrifugation, and the CD14^+^ population was sorted using CD14 MicroBeads (Miltenyi) following the manufacturer's indications. Cells were plated at 2 × 10^5^ cells/cm^2^ and cultured in an MLR medium supplemented with 20 ng/ml of macrophage colony-stimulating factor (M-CSF) (Miltenyi). After 7 days of culture, macrophages were activated with 100 ng/ml of lipopolysaccharides (LPS) and cultured alone, with naïve hAD-MSC or pretreated hAD-MSC at a ratio of 1 : 10 for 24 h. To assess macrophage polarization, cells were harvested using Versene (Gibco) and incubated with 100 *μ*M of Fc block (BD Bioscience) following the manufacturer's indications. Then, cells were stained using a cell viability marker (Life Technologies) and antibodies against CD86 (clone 2331 Fun-1), CD163 (clone GHI/61) (BD Bioscience), CD68 (clone Y1/82A), CD206 (clone 15-2), and HLA-DR (clone L243) (BioLegend, San Diego, California, USA). Data were acquired with a BD FACSCanto and analyzed using the FlowJo software (v.10) (TreeStar Inc.).

### 2.9. Indoleamine 2,3-Dioxygenase (IDO) Activity

In order to quantify the IDO activity, we analyzed the tryptophan-to-kynurenine conversion using a photometric detection method. 60 *μ*l of cell supernatant (conditioned medium) was pipetted to a 96-well culture plate, and 30 *μ*l of 30% trichloroacetic acid solution (Sigma) was added for 30 min at 50°C. After a centrifugation step, 75 *μ*l of the samples were added to 75 *μ*l of freshly prepared Ehrlich's solution (Sigma, MO, USA), and photoabsorbance was read at 450 nM on Multiskan plate reader (Thermo Fisher).

### 2.10. Statistical Analysis

GraphPad Prism 7.0v software (GraphPad, CA, USA) was used for the presentation of the results and statistical analysis. All over the manuscript, results were expressed as the mean ± SEM. The statistical analysis was performed using the nonparametric Kruskal-Wallis followed by Dunn's post hoc test for multiple comparisons. For comparison between the two groups, the Mann–Whitney *U* or Wilcoxon (IDO activity) tests were used. Statistical significance was noted as ns for *p* > 0.05, ^∗^ for *p* < 0.05, ^∗∗^ for *p* < 0.01, ^∗∗∗^ for *p* < 0.001, and ^∗∗∗∗^ for *p* < 0.0001.

## 3. Results

### 3.1. Screening PPAR*β*/*δ* Interactions to Develop an Interfering Peptide

**Figure 1:**
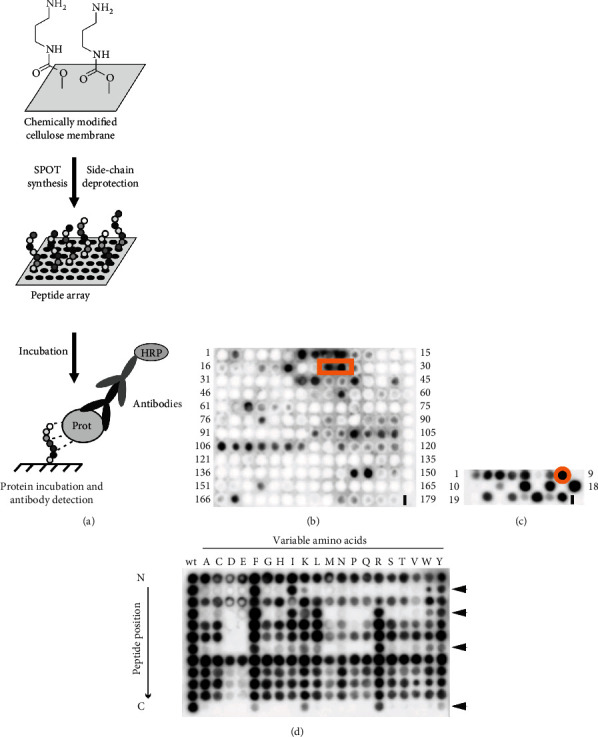
Determining a PPAR*β*/*δ* interfering peptide by SPOT synthesis. (a) General concept of the SPOT synthesis to synthesize membrane-bound peptides and of their protein incubation followed by antibody detection. (b) TF65 PepScan: primary sequence of the TF65 protein was dissected in overlapping peptides (15-mer with a shift of 3 amino acids) which were synthesized on the chemically modified cellulose membrane. Peptides were incubated with the His-tagged PPAR*β*/*δ* protein revealing a potent epitope corresponding to the spots n°24-25 (orange square). (c) Length analysis of the 18-mer peptide (spots n°24-25, cut at N- and C-terminus) showed the minimal peptide length (12-mer peptide of spot n°8, orange circle) required for TF65 binding. (d) Substitutional analysis of the 12-mer PP11 peptide revealed four key positions corresponding to the amino acids of isoleucine, leucine, lysine, and arginine (see arrows).

It is known that PPAR*β*/*δ* can bind to proteins including nuclear factor NF-*κ*B TF65 subunit (also called transcription factor p65, TF65) and attenuate NF-*κ*B-dependent signaling [13]. However, to our best knowledge, no structural information showing the interface of this interaction is published so far. To screen PPAR*β*/*δ*:NF-*κ*B TF65 interaction, we used the SPOT technology allowing the parallel synthesis of cellulose-bound peptide libraries for the screening of protein-protein interaction (Figure 1(a)).

**Figure 2:**
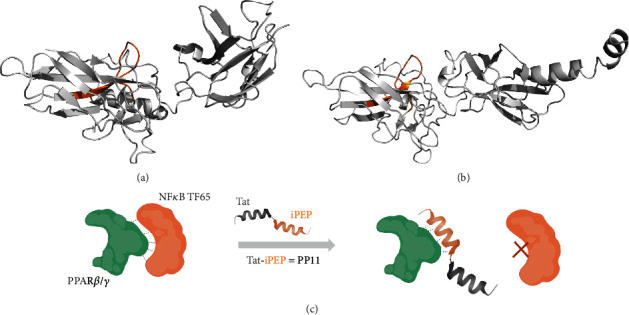
Visualization of the PP11 sequence with the TF65 structure. NF-*κ*B TF65 structures were extracted from the TF65:IRF-7:IRF-3:DNA complex (PDB #2O61) [15] (a) and from the I*κ*B*α*:TF65 complex (PDB #1NFI) [14] (b). The PP11 peptide sequence situated in an accessible loop is highlighted in orange. Both 3D models were made using PyMol (c). Schematic representation of the PP11 peptide interaction mode: the PP11 peptide acts as a mimic of the NF-*κB* TF65 subunit and prevents PPAR*β*/*δ* interaction to the NF-*κ*B TF65 subunit (scheme partially realized with BioRender, https://www.biorender.com).

First, a PepScan analysis was performed by dissecting the primary sequence of TF65 in overlapping peptides to determine the NF-*κ*B TF65 epitope involved in the interaction with PPAR*β*/*δ* (Figure 1(b)). Because a direct correlation between signal intensity and affinity could not be performed using this technology, we set an arbitrary threshold (values > 100,000) to determine “binders” (Supplementary materials, Table S1). Many potent epitopes were detected after PPAR*β*/*δ* protein incubation. To select the more potent one that is able to interrupt PPAR*β*/*δ*:TF65 interaction, we used the two published crystal structures of NF-*κ*B TF65 [14, 15] to evaluate the position of the different epitopes (Figure 2). The epitope situated in one of the TF65 loops corresponding to the spots n°24 to 25 (70-GTVRISLVTKDPPHRPHP-87) of the PepScan was selected based on its accessibility for protein-protein interaction (red square in Figure 1(b), red part in the two structures of Figure 2).

Afterward, a length analysis was performed on this 18-mer peptide to define the optimal length for PPAR*β*/*δ* binding by cutting successively the N- and C-terminal regions of the peptide. After PPAR*β*/*δ* incubation, the 12-mer sequence of spot n°8 (73-RISLVTKDPPHR-84) was determined as the peptide having one of the highest signal intensities (Figure 1(c) and Supplementary materials, Table S2). Finally, this 12-mer peptide was evaluated by substitutional analysis (SubAna, amino acid mutation at each sequence position) to determine the potent key positions of PPAR*β*/*δ*:TF65 interaction (Figure 1(d) and Supplementary materials, Table S3). The first column of the SubAna corresponds to the wild-type (wt) sequence and the following column to the exchanged amino acids. The SubAna revealed 4 key positions of the peptide: isoleucine, leucine, lysine, and arginine (RISLVTKDPPHR) which could not be arbitrarily replaced. In many cases, SubAnas with a clear and defined pattern of key positions will demonstrate a specific protein-peptide interaction [16–18].

The determined 12-mer peptide will act as a mimic of the NF-*κ*B TF65 subunit and prevent PPAR*β*/*δ* interaction to the NF-*κ*B TF65 subunit. To evaluate its cellular activity, the found decoy peptide was coupled to the commonly used cell-penetrating peptide Tat [19] to allow cellular internalization. Tat conjugated to the PPAR*β*/*δ*-interfering peptide is afterward called PP11 (Figure 2(c)).

### 3.2. PP11 Peptide Downregulates the Expression Level of PPAR*β*/*δ* Target Genes without Affecting those of PPAR*α* or PPAR*γ*

**Figure 3:**
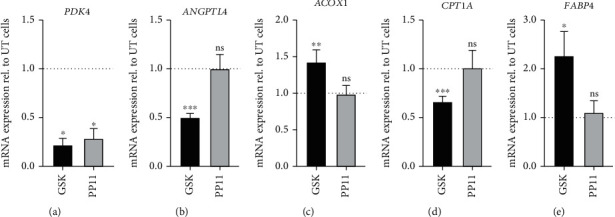
Expression profile of PPAR*β*/*δ*, PPAR*α*, and PPAR*γ* target genes in hAD-MSC inactivated for PPAR*β*/*δ*. (a, b) Comparison of the expression levels of PPAR*β*/*δ* target genes (*PDK4* and *ANGPTL4*) between untreated (UT) hAD-MSC (dashed line), GSK3787-pretreated hAD-MSC (GSK), and PP11-pretreated hAD-MSC (PP11). *PDK4* (a) and *ANGPTL4* (b) expressions were normalized to housekeeping gene (*RPS9*) expression. mRNA expression levels in hAD-MSC treated during 24 h with 0.1 *μ*M of GSK3787 (GSK) or 0.1 *μ*M of PP11 were standardized to expression in nonpretreated hAD-MSC (dashed line), and results are expressed using the 2^−ΔΔCt^ method. (c, d) Comparison of the expression levels of PPAR*α* target genes (*ACOX1* and *CPT1A*) between untreated hAD-MSC (dashed line), GSK3787-pretreated hAD-MSC (GSK), and PP11-pretreated AD-MSC (PP11). *ACOX1* (c) and *CPT1A* (d) expressions were normalized to housekeeping gene (*RPS9*) expression. mRNA expression levels in hAD-MSC treated during 24 h with 0.1 *μ*M of GSK3787 (GSK) or 0.1 *μ*M of PP11 were standardized to expression in untreated hAD-MSC (dashed line), and results are expressed using the 2^−ΔΔCt^ method. (e) Comparison of the expression level of PPAR*γ* target gene (*FABP4*) between untreated hAD-MSC (dashed line), GSK3787-pretreated hAD-MSC (GSK), and PP11-pretreated hAD-MSC (PP11). *FABP4* expression was normalized to housekeeping gene (*RPS9*) expression. mRNA expression levels in hAD-MSC treated during 24 h with GSK3787 (GSK, 0.1 *μ*M) or PP11 (0.1 *μ*M) were standardized to expression in untreated hAD-MSC (dashed line), and results are expressed using the 2^−ΔΔCt^ method. These results were obtained with hAD-MSC from 4 donors (*N* = 4) in 2 or 3 independent experiments. The statistical analysis was performed using the Mann–Whitney *U* test. Statistical significance was noted as ns for *p* > 0.05, ^∗^ for *p* < 0.05, ^∗∗^ for *p* < 0.01, and ^∗∗∗^ for *p* < 0.001.

To determine the specificity of PP11, we assessed the expression level of *PDK4* and *ANGPTL4* and PPAR*β*/*δ* target genes, as well as the expression levels of *ACOX1* and *CPT1A*, PPAR*α* target genes, and *FABP4*, a PPAR*γ* target gene in untreated hAD-MSC compared to human hAD-MSC pretreated with either GSK3787 (a PPAR*β*/*δ* antagonist) or PP11. Using RT-qPCR, we observed that the 24 h pretreatment of hAD-MSC with 0.1 *μ*M of PP11 or GSK3787 significantly reduced the expression level of *PDK4* (Figure 3(a)) as compared to untreated hAD-MSC (dashed line). In contrast, the *ANGPTL4* expression level was affected only after the pretreatment with GSK3787 but not with PP11, as compared to untreated hAD-MSC (Figure 3(b)). Then, we assessed the expression levels of PPAR*α* target genes and found that the 24 h pretreatment of hAD-MSC with 0.1 *μ*M of PP11 did not modify the expression level of the two PPAR*α* target genes tested, whereas 0.1 *μ*M GSK3787 significantly increased *ACOX1* (Figure 3(c)) and decreased *CPT1A* (Figure 3(d)). Similarly, while PP11 pretreatment did not modulate the *FABP4* expression level, a PPAR*γ* target genes, GSK3787 significantly increased it (Figure 3(e)). Altogether, these results indicate the specificity of the newly synthesized PP11 peptide which reduces specifically the expression level of *PDK4*, a PPAR*β*/*δ* target gene, but not the expression levels of PPAR*α* or PPAR*γ* target genes.

### 3.3. Human AD-MSC Pretreated with PP11 Peptide Exhibit Enhanced Immunoregulatory Properties

**Figure 4:**
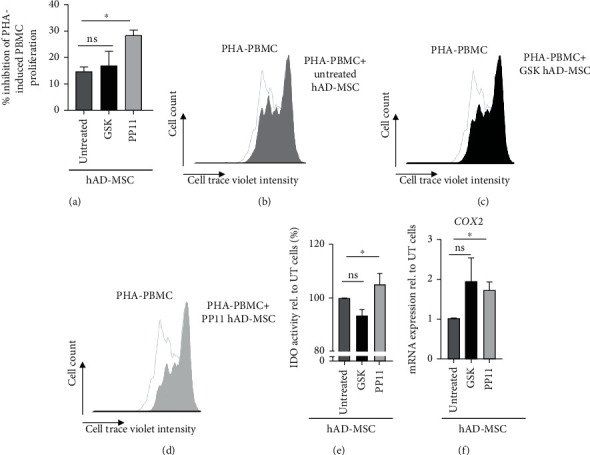
PP11 peptide significantly enhanced hAD-MSC immunosuppressive properties. (a–d) Effect of PPAR*β*/*δ* activity modulation on hAD-MSC immunosuppressive properties. PBMC stained with CTV were collected and analyzed by FACS to determine their proliferation rate (b–d) and calculate the percentage of inhibition (a) of PHA-activated PBMC cocultured in the presence of untreated hAD-MSC, hAD-MSC pretreated with GSK3787 (GSK hAD-MSC), or hAD-MSC pretreated with PP11 (PP11 hAD-MSC). (e, f) Impact of PPAR*β*/*δ* inactivation on immunomodulatory mediators. (e) IDO activity determined by a spectrophotometric assay for kynurenine in supernatant of cocultured hAD-MSC:PBMC. Nontreated (Untreated), GSK3787 (GSK), or PP11- (PP11-) pretreated hAD-MSC were cocultured with PHA-activated PBMC for 96 h. The kynurenine concentration in the presence of pretreated cells is expressed relative to that measured in the presence of untreated cells (%). (f) *COX2* mRNA expression level in hAD-MSC was pretreated either with GSK3787 (GSK) or PP11 peptide (PP11), compared to untreated hAD-MSC. *COX2* expression level was normalized to housekeeping gene (*RPS9*) expression. mRNA expression in treated cells was standardized to expression in untreated hAD-MSC, and results are expressed using the 2^−ΔΔCt^ method. The results for proliferation inhibition were obtained with hAD-MSC from 4 donors (*N* = 4). The statistical analysis was performed using the Mann–Whitney *U* test. Statistical significance was noted as ns for *p* > 0.05 and ^∗^ for *p* < 0.05. The results for IDO activity were obtained with hAD-MSC from 3 donors (*N* = 3) on 2 independent experiments. The statistical analysis was performed using the Wilcoxon test. Statistical significance was noted as ns for *p* > 0.05 and ^∗^ for *p* < 0.05. The results for *COX2* expression were obtained with hAD-MSC from 3 donors (*N* = 3) on 2 independent experiments. The statistical analysis was performed using the Mann–Whitney *U* test. Statistical significance was noted as ns for *p* > 0.05 and ^∗^ for *p* < 0.05.

We investigated the role of PPAR*β*/*δ* modulation using either the PP11 peptide or GSK3787 on hAD-MSC immunoregulatory functions. To that end, hAD-MSCs were pretreated with 0.1 *μ*M of either PP11 or GSK3787 for 24 h before being cocultured with phytohemagglutinin- (PHA-) activated PBMC at a ratio of 1 : 10 for 4 days. First, we confirmed the immunosuppressive properties of hAD-MSC on CellTrace Violet- (CTV-) labeled PBMC activated with PHA (Figures 4(a) and 4(b)). Although the inhibition of PPAR*β*/*δ* activity using the GSK3787 antagonist tended to increase the capacity of hAD-MSC to inhibit the proliferation of PHA-activated PBMC, no significant change in hAD-MSC immunoregulatory potential was observed compared to untreated hAD-MSC (Figures 4(a) and 4(c)). In contrast, the pretreatment of hAD-MSC with PP11 peptide significantly enhanced their immunoregulatory properties compared to their untreated counterpart (Figures 4(a) and 4(d)). Altogether, these results suggest that the PPAR*β*/*δ*-mediated repression of TF65, lifted/removed by the PP11 peptide, is pivotal for hAD-MSC immunosuppressive functions.

Then, we assessed the effect of PPAR*β*/*δ* activity modulation on the indoleamine 2,3-dioxygenase (IDO) activity, involved in MSC immunoregulatory properties [20], in hAD-MSC cocultured with PHA-activated PBMC. Compared to untreated hAD-MSC, we showed that hAD-MSC treated with GSK3787 exhibited a significantly lower level of IDO activity while hAD-MSC pretreated with PP11 peptide had a significantly higher IDO activity (Figure 4(e)). Moreover, we evaluated the expression level of the cyclooxygenase-2 (*COX2*) gene, another MSC immunoregulatory mediator [21] in hAD-MSC pretreated with GSK3787 and PP11. Using RT-qPCR, we observed an increased mRNA expression level of *COX2* in response to the two pretreatments as compared to untreated hAD-MSC, but a significant increase was obtained only with the PP11 peptide (Figure 4(f)).

### 3.4. Human AD-MSC Pretreated with PP11 Peptide Exhibit an Enhanced Immunoregulatory Property on Macrophages

**Figure 5:**
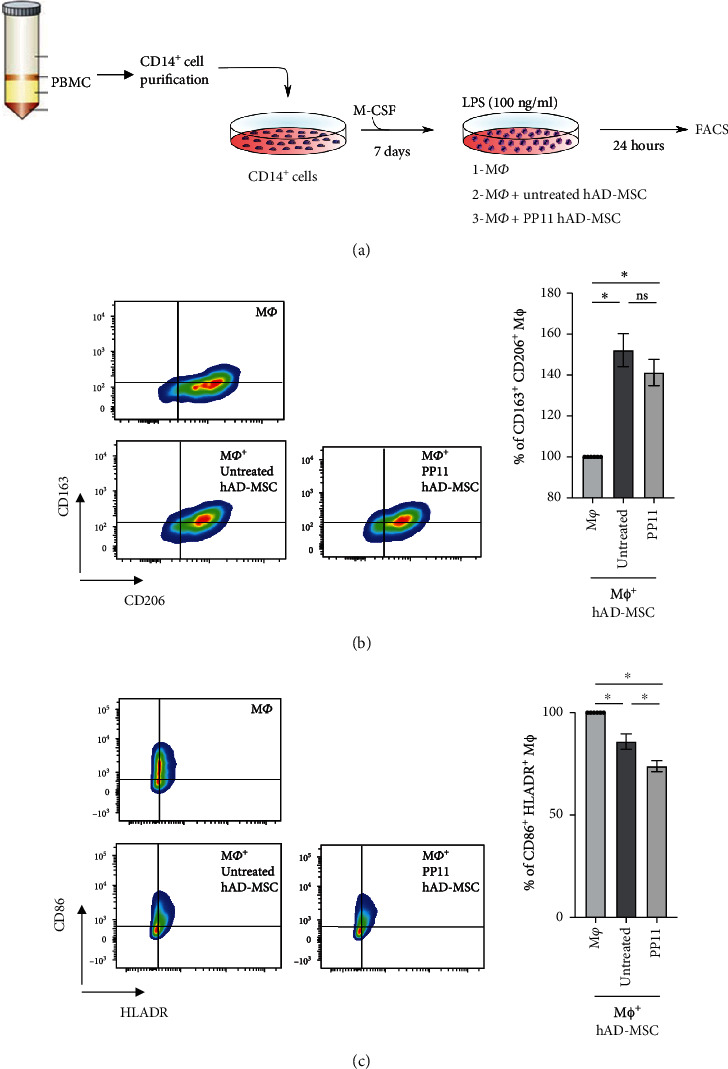
Role of the interaction between PPAR*β*/*δ* and NF*-κ*B TF65 subunit in hAD-MSC immunoregulatory property on macrophages. (a) Experimental design of human macrophage differentiation and activation. CD14^+^ population was sorted from human PBMC using CD14 MicroBeads (Miltenyi) and cultured with 20 ng/ml of M-CSF during 7 days. Then, the resulting macrophages were activated with 100 ng/ml of lipopolysaccharides (LPS) and cultured alone (M*ϕ*), naïve hAD-MSC (M*ϕ*+ untretated hAD-MSC) or PP11-pretreated hAD-MSC (M*ϕ*+PP11 hAD-MSC) at ratio 1 : 10 for 24 h. Cells were harvested to analyze macrophage phenotype by FACS. (b) Representative dot plots showing the M2-like macrophage population. Macrophages were analyzed for the expression profile of M2 markers including CD163 and CD206 to determine the percentage of M2-like macrophages CD163^+^CD206^+^ when LPS-activated macrophages were cultured alone (M*ϕ*), in the presence of naïve hAD-MSC (M*ϕ*+ untretated hAD-MSC) or hAD-MSC pretreated with PP11 peptide (M*ϕ*+PP11 hAD-MSC). (c) Representative dot plots showing the M1-like macrophage population. Macrophages were analyzed for the expression profile of M1 markers including CD86 and HLADR to determine the percentage of M1-like macrophages CD86^+^HLADR^+^. The statistical analysis was performed using the nonparametric Kruskal-Wallis followed by Dunn's post hoc test for multiple comparisons. The data were obtained with hAD-MSC from 3 donors (*N* = 3) on 2 independent experiments. Statistical significance was noted as ns for *p* > 0.05 and ^∗^ for *p* < 0.05.

Finally, we assessed the effect of the modulation of PPAR*β*/*δ* activity on MSC immunoregulatory functions focusing on macrophages that play a pivotal role in OA pathogenesis. MSC regulates the macrophage response by inducing M1-like macrophage polarization toward a M2-like phenotype in a COX2-dependent manner [22–24]. We thus wondered whether the modulation of PPAR*β*/*δ* activity in human hAD-MSC using the PP11 peptide could modify their capacity to control the macrophage response. To address that hypothesis, we performed coculture experiments with M1-like macrophages and hAD-MSC pretreated with PP11 peptide (Figure 5(a)). First, we confirmed that the coculture of hAD-MSC with M1-like macrophages (M*ϕ*+hAD-MSC) significantly increased the percentage of M2-like macrophages positive for CD163 and CD206 (Figure 5(b)) and decrease the percentage of M1-like macrophages positive for CD86 and HLA-DR, as compared to M1-like macrophages cultured alone (M*ϕ*) (Figure 5(c)). When cocultured with hAD-MSC pretreated with PP11 peptide, the percentage of M2-like macrophages positive for CD163 and CD206 was significantly increased compared to macrophages cultured alone but no significant difference was observed compared to macrophages cultured with untreated hAD-MSC (Figure 5(b)). In contrast, while both untreated hAD-MSC and PP11-treated hAD-MSC significantly decreased the percentage of M1-like macrophages positive for CD86 and HLA-DR, PP11-treated hAD-MSC exhibited a more potent immunoregulatory property (Figure 5(c)). This result suggests that the repression of PPAR*β*/*δ* interaction with TF65, a subunit of the NF-*κ*B transcription factor complex, promoting the nuclear translocation of NF-*κ*B in hAD-MSC, enhances the immunoregulatory potential of hAD-MSC on proinflammatory M1-like macrophages.

## 4. Discussion

In the present study, we design and synthesize a specific inhibitor of PPAR*β*/*δ*:NF-*κ*B TF65 interaction, PP11, and demonstrate in hAD-MSC its potent effect in controlling PPAR*β*/*δ* activity without affecting the expression levels of the target genes of other PPAR family members. Human AD-MSC treated with PP11 exhibit enhanced capacity to inhibit the proliferation of activated PBMC and to regulate the macrophage response associated with an increased IDO activity.

Deciphering protein-protein interactions (PPIs) involved in various diseases is an interesting strategy to engineer interfering peptides. Indeed, a complex and dynamic network of PPIs regulates most cellular functions and in particular signaling cascades leading to cell proliferation or death. Specific inhibitors of PPIs are thus emerging as a new class of drugs with many potential applications in basic and clinical research. Unfortunately, PPIs have often been considered nondruggable for several reasons. First, the PPI architecture is often not sufficiently known to allow the rational design of inhibitors. Second, and more importantly, PPIs occur through large, flat surfaces often without hydrophobic pockets (unlike receptor-ligand or enzyme-substrate complexes), making conventional small molecule drugs often ineffective. In contrast, interfering peptides (10 to 15-mer) that can interact with PPIs are interesting therapeutic molecules because of their potential to inactivate or activate a signaling cascade. Over the past, different screening strategies were developed to determine interfering peptides such as yeast two-hybrid, phage display, or the SPOT synthesis used here [25, 26].

Due to their good solubility, favorable pharmacokinetic profile, low toxicity/mitogenicity, and infinite possibility to improve their binding stability/affinity by modifications, peptides are considered as interesting therapeutic molecules [27]. Therefore, the pharmaceutical industry considered, nowadays, peptides as therapeutics as evidenced by more than 60 peptide drugs approved by the Food Drug Administration and a continuously growing market over the last two decades [28].

To develop an interfering peptide of the complex formed between PPAR*β*/*δ* and the nuclear factor NF-*κ*B TF65 subunit, we have synthesized and screened different peptide libraries by SPOT synthesis. This method allows us to elucidate the binding region of the NF-*κ*B TF65 subunit important for PPAR*β*/*δ* interaction (70-GTVRISLVTKDPPHRPHP-87, 18-mer), to reduce this epitope to a 12-mer peptide and to determine the key positions (73-RISLVTKDPPHR-84). By coupling the decoy peptide to the cell-penetrating peptide Tat (10-mer), the cellular internalization of this conjugate, called afterward PP11, was ensured (data not shown). Within the cell, PP11 will interact as peptidyl antagonist by mimicking the NF-*κ*B TF65 subunit, which in turn could have an impact of PPAR*β*/*δ* downstream pathways.

Pyruvate dehydrogenase kinase 4 (PDK4) is expressed by adipocytes, and increased expression of PDK4 is associated with inhibited glucose uptake [29]. PDK4 acts as an inhibitor of pyruvate oxidation and is involved in the change of cellular metabolism [30]. The activation of the three main isoforms of the PPAR family including PPAR*α*, PPAR*β*/*δ*, and PPAR*γ* increases PDK4 expression which involves a coordinated metabolic transfer from glucose to fatty acids as the primary energy fuel [30]. Here, we show that PP11 as well as GSK3787, a potent antagonist of PPAR*β*/*δ*, significantly decreased the expression of *PDK4* in human AD-MSC without altering the expression levels of PPAR*α* and PPAR*γ* target genes. This result not only demonstrates that PP11 is as effective as GSK3787 in regulating the expression of a PPAR*β*/*δ* target gene but also that it is more specific than a commercially available antagonist. This decreased expression level of PDK4 induced by PP11 paralleled the enhanced immunoregulatory properties of human AD-MSC on PHA-activated PBMC. Based on our previous work, showing that the proglycolytic switch of MSC mediated by oligomycin significantly enhanced their immunoregulatory properties *in vitro* [31]; it is tempting to speculate that AD-MSC pretreatment with PP11 induces a metabolic transfer from fatty acids as the primary energy fuel to glucose in cells. Of course, this hypothesis will require further investigations.

Angiopoietin-like 4 protein (ANGPTL4) is a member of the angiopoietin-like family of proteins structurally related to factors modulating angiogenesis first identified as an adipokine exclusively involved in lipid metabolism [32]. ANGPTL4 has since been shown to be involved in cell differentiation, tumorigenesis, energy and glucose homeostasis, lipid metabolism, wound healing, and inflammation regulation [33], *ANGPTL4* transcriptional regulation can be regulated by nutritional and hormonal conditions as well as several transcription factors, including PPAR*α*, PPAR*β*/*δ*, PPAR*γ* [33], and HIF1*α* [34]. Unexpectedly, while the treatment of hAD-MSC with a PPAR*β*/*δ* antagonist, GSK3787, significantly reduced the expression of *ANGPTL4*, the treatment of the cells with PP11 did not. This could be explained by the fact that the transcriptional regulation of *ANGPTL4* is mediated by different factors other than PPAR*β*/*δ* that could be present in the culture medium of hAD-MSCs or expressed by the cells themselves. Further investigations are needed to clarify that point and confirm the specificity of PP11 for PPAR*β*/*δ* and its targets. Although puzzling, this result is very intriguing and could explain the increased immunoregulatory effect obtained with hAD-MSC treated with PP11 as compared to GSK3787 in the *in vitro* functional tests. Indeed, we showed that PP11-interfering peptide blocks PPAR*β*/*δ* activity without affecting *ANGPTL4* expression in human AD-MSC. Moreover, the treatment of hAD-MSC with PP11 but not GSK3787 significantly increased their IDO activity compared to the untreated cells and increased the expression level of *COX2* that regulates the production of prostaglandin E2 (PGE2), well-known mediators of MSC immunoregulatory properties. Altogether, these effects mediated by PP11 on *ANGPTL4* and *COX2* expression and IDO activity in human AD-MSC might explain their enhanced immunoregulatory properties. While the role of COX2 and IDO on MSC immunosuppressive properties was described a while ago, the role of *ANGPTL4* is more recent. Indeed, MSC overexpressing *COX2* more potently inhibited the activation and proliferation of PBMC [21, 35]. Regarding IDO, it is one of the most critical factors in human MSC-directed immunoregulation [35, 36]. For the *ANGPTL4* part, it was more recently shown that in conditions of coculture with macrophages, MSC markedly expressed *ANGPTL4* to dampen macrophage polarization toward the proinflammatory phenotype. MSCs lacking *ANGPTL4* were not successful in inhibiting the inflammatory phenotype of macrophages [37]. Altogether, these results suggest that the enhanced immunoregulatory properties of human AD-MSC treated with PP11 or GSK3787 compared to naïve MSC or GSK3787 are associated with the higher expression of *ANGPTL4* and *COX2* and the higher activity of IDO. Further studies should be investigated to confirm the pivotal role of *ANGPTL4* induced by PP11 on MSC immunosuppressive properties.

In this study, we have developed PP11, an interfering peptide of the complex formed between PPAR*β*/*δ* and TF65 subunit of the nuclear factor NF-*κ*B, that plays a pivotal role in MSC immunosuppressive properties. Although we demonstrated that PP11 enhanced MSC immunosuppressive properties, the effect of PP11 on NF-*κ*B activity in human AD-MSC and on the regulation of NF-*κ*B target genes remains to be studied. Blocking the interaction between PPAR*β*/*δ* and NF-*κ*B TF65 subunit may also impact NF-*κ*B TF65 pathway itself resulting in (1) a more rapid proteasomal degradation, (2) a potent higher interaction with other cytoplasmic or nuclear proteins, and (3) an exceeding formation of various NF-*κ*B subunit dimer combinations inducing a heterogeneous regulation of NF-*κ*B target genes. However, the effect of PP11 on NF-*κ*B TF65 signaling pathways is likely to be the subject of another manuscript.

## 5. Conclusions

In conclusion, we designed and synthesized an interfering peptide that specifically blocks PPAR*β*/*δ* activity and enhances MSC immunoregulatory properties *in vitro* both on activated PBMC and proinflammatory macrophages. These promising results are an important step to better understand the MSC immunoregulatory properties even if additional biochemical experiments will be necessary to highlight the PP11 effect on the PPAR*β*/*δ*:NF-*κ*B TF65 complex. Our study raises the possibility that PP11 will be a more specific PPAR*β*/*δ* antagonist than the commercially available drugs.

## Data Availability

Data are available on request through the authors (Gautier Tejedor and Farida Djouad).

## Supplementary Materials

Supplementary Materials Table S1: sequences of the TF65 PepScan with the corresponding signal intensities (SI). Table S2: sequences of the length analysis of the peptide GTVRISLVTKDPPHRPHP (spot 24-25 of Table S1) with the corresponding signal intensities. Table S3: sequences of the substitutional analysis of the peptide RISLVTKDPPHR (spot 8 of Table S2) with the corresponding signal intensities.
