# Supplementary material for: PPARβ/δ-Interfering Peptide Enhanced Mesenchymal Stromal Cell Immunoregulatory Properties
Source: Stem Cells Int. 2022 Dec 13;2022:5494749. doi: 10.1155/2022/5494749 (PMC9767714; doi:10.1155/2022/5494749)
Supplement: Supplementary Materials — Table S1: sequences of the TF65 PepScan with the corresponding signal intensities (SI). Table S2: sequences of the length analysis of the peptide GTVRISLVTKDPPHRPHP (spot 24-25 of Table S1) with the corresponding signal intensities. Table S3: sequences of the substitutional analysis of the peptide RISLVTKDPPHR (spot 8 of Table S2) with the corresponding signal intensities. [file 5494749.f1.pdf]

PPARβ/δ interfering peptide enhanced mesenchymal stromal cell immunoregulatory properties

Gautier Tejedor[1,2], Prisca Boisguerin[3], Éric Vivès[3], Christian Jorgensen[1,4], Jérôme Guicheux[5], Claire Vinatier[5], Claire Gondeau[2] and Farida Djouad[1*]

[1]IRMB, Univ Montpellier, INSERM, Montpellier, France; [2]MedXCell Science SAS, Montpellier, France; [3]PhyMedExp, Univ Montpellier, INSERM, CNRS, Montpellier, France; [4]CHU Montpellier, Montpellier, F-34295, France; [5]Nantes Université, Oniris, CHU Nantes, INSERM, Regenerative Medicine and Skeleton, RMeS, UMR 1229, F-44000 Nantes, France.

Corresponding author:

Farida Djouad, Inserm U 1183, IRMB, Hôpital Saint-Eloi, 80 avenue Augustin Fliche, 34295 Montpellier cedex 5, France. Tel: 33 (0) 4 67 33 04 75 – Fax: 33 (0) 4 67 33 01 13 –

E-mail: farida.djouad@inserm.fr

Table S1: Sequences of the TF65 PepScan with the corresponding signal intensities (SI)

Spot Nb	Sequences	Signal intensities
**1**	MDELFPLIFPAEPAQ	4,353
**2**	LFPLIFPAEPAQASG	65,903
**3**	LIFPAEPAQASGPYV	3,555
**4**	PAEPAQASGPYVEII	2,851
**5**	PAQASGPYVEIIEQP	3,862
**6**	ASGPYVEIIEQPKQR	22,805
**7**	PYVEIIEQPKQRGMR	**172,781**
**8**	EIIEQPKQRGMRFRY	**126,029**
**9**	EQPKQRGMRFRYKCE	**102,462**
**10**	KQRGMRFRYKCEGRS	**149,432**
**11**	GMRFRYKCEGRSAGS	41,231
**12**	FRYKCEGRSAGSIPG	31,791
**13**	KCEGRSAGSIPGERS	3,087
**14**	GRSAGSIPGERSTDT	2,736
**15**	AGSIPGERSTDTTKT	1,707
**16**	IPGERSTDTTKTHPT	14,576
**17**	ERSTDTTKTHPTIKI	13,651
**18**	TDTTKTHPTIKINGY	6,289
**19**	TKTHPTIKINGYTGP	41,083
**20**	HPTIKINGYTGPGTV	27,921
**21**	IKINGYTGPGTVRIS	**153,746**
**22**	NGYTGPGTVRISLVT	8,574
**23**	TGPGTVRISLVTKDP	19,980
**24**	GTVRISLVTKDPPHR	**132,841**
**25**	RISLVTKDPPHRPHP	**171,742**
**26**	LVTKDPPHRPHPHEL	45,465
**27**	KDPPHRPHPHELVGK	4,974
**28**	PHRPHPHELVGKDCR	1,439
**29**	PHPHELVGKDCRDGF	1,667
**30**	HELVGKDCRDGFYEA	2,013
**31**	VGKDCRDGFYEAELC	1,368
**32**	DCRDGFYEAELCPDR	2,362
**33**	DGFYEAELCPDRCIH	2,913
**34**	YEAELCPDRCIHSFQ	2,781
**35**	ELCPDRCIHSFQNLG	3,358
**36**	PDRCIHSFQNLGIQC	8,220
**37**	CIHSFQNLGIQCVKK	97,412
**38**	SFQNLGIQCVKKRDL	**125,671**
**39**	NLGIQCVKKRDLEQA	44,329
**40**	IQCVKKRDLEQAISQ	41,916
**41**	VKKRDLEQAISQRIQ	**119,520**
**42**	RDLEQAISQRIQTNN	9,120
**43**	EQAISQRIQTNNNPF	2,739
**44**	ISQRIQTNNNPFQVP	56,351
**45**	RIQTNNNPFQVPIEE	2,714

**46**	TNNNPFQVPIEEQRG	1,902
**47**	NPFQVPIEEQRGDYD	3,205
**48**	QVPIEEQRGDYDLNA	4,210
**49**	IEEQRGDYDLNAVRL	8,865
**50**	QRGDYDLNAVRLCFQ	6,172
**51**	DYDLNAVRLCFQVTV	2,620
**52**	LNAVRLCFQVTVRDP	5,232
**53**	VRLCFQVTVRDPSGR	25,723
**54**	CFQVTVRDPSGRPLR	22,699
**55**	VTVRDPSGRPLRLPP	98,511
**56**	RDPSGRPLRLPPVLS	13,034
**57**	SGRPLRLPPVLSHPI	6,777
**58**	PLRLPPVLSHPIFDN	21,146
**59**	LPPVLSHPIFDNRAP	13,636
**60**	VLSHPIFDNRAPNTA	6,045
**61**	HPIFDNRAPNTAELK	3,015
**62**	FDNRAPNTAELKICR	21,888
**63**	RAPNTAELKICRVNR	**105,962**
**64**	NTAELKICRVNRNSG	37,451
**65**	ELKICRVNRNSGSCL	22,405
**66**	ICRVNRNSGSCLGGD	18,298
**67**	VNRNSGSCLGGDEIF	3,582
**68**	NSGSCLGGDEIFLLC	3,439
**69**	SCLGGDEIFLLCDKV	5,623
**70**	GGDEIFLLCDKVQKE	6,379
**71**	EIFLLCDKVQKEDIE	19,211
**72**	LLCDKVQKEDIEVYF	6,455
**73**	DKVQKEDIEVYFTGP	3,630
**74**	QKEDIEVYFTGPGWE	3,139
**75**	DIEVYFTGPGWEARG	3,309
**76**	VYFTGPGWEARGSFS	13,971
**77**	TGPGWEARGSFSQAD	2,549
**78**	GWEARGSFSQADVHR	6,064
**79**	ARGSFSQADVHRQVA	64,035
**80**	SFSQADVHRQVAIVF	5,493
**81**	QADVHRQVAIVFRTP	3,797
**82**	VHRQVAIVFRTPPYA	7,981
**83**	QVAIVFRTPPYADPS	4,635
**84**	IVFRTPPYADPSLQA	42,450
**85**	RTPPYADPSLQAPVR	11,722
**86**	PYADPSLQAPVRVSM	4,754
**87**	DPSLQAPVRVSMQLR	31,549
**88**	LQAPVRVSMQLRRPS	40,550
**89**	PVRVSMQLRRPSDRE	43,443
**90**	VSMQLRRPSDRELSE	3,108
**91**	QLRRPSDRELSEPME	2,219
**92**	RPSDRELSEPMEFQY	2,367
**93**	DRELSEPMEFQYLPD	2,733

**94**	LSEPMEFQYLPDTDD	2,280
**95**	PMEFQYLPDTDDRHR	3,852
**96**	FQYLPDTDDRHRIEE	3,901
**97**	LPDTDDRHRIEEKRK	4,206
**98**	TDDRHRIEEKRKRTY	56,552
**99**	RHRIEEKRKRTYETF	32,050
**100**	IEEKRKRTYETFKSI	48,995
**101**	KRKRTYETFKSIMKK	**148,090**
**102**	RTYETFKSIMKKSPF	68,598
**103**	ETFKSIMKKSPFSGP	85,420
**104**	KSIMKKSPFSGPTDP	81,525
**105**	MKKSPFSGPTDPRPP	3,032
**106**	SPFSGPTDPRPPPRR	**137,061**
**107**	SGPTDPRPPPRRIAV	88,447
**108**	TDPRPPPRRIAVPSR	**117,857**
**109**	RPPPRRIAVPSRSSA	84,283
**110**	PRRIAVPSRSSASVP	64,694
**111**	IAVPSRSSASVPKPA	81,654
**112**	PSRSSASVPKPAPQP	54,794
**113**	SSASVPKPAPQPYPF	4,471
**114**	SVPKPAPQPYPFTSS	6,561
**115**	KPAPQPYPFTSSLST	59,678
**116**	PQPYPFTSSLSTINY	30,876
**117**	YPFTSSLSTINYDEF	29,641
**118**	TSSLSTINYDEFPTM	41,159
**119**	LSTINYDEFPTMVFP	44,899
**120**	INYDEFPTMVFPSGQ	2,707
**121**	DEFPTMVFPSGQISQ	2,781
**122**	PTMVFPSGQISQASA	3,956
**123**	VFPSGQISQASALAP	10,428
**124**	SGQISQASALAPAPP	3,443
**125**	ISQASALAPAPPQVL	2,435
**126**	ASALAPAPPQVLPQA	2,011
**127**	LAPAPPQVLPQAPAP	1,445
**128**	APPQVLPQAPAPAPA	1,462
**129**	QVLPQAPAPAPAPAM	2,902
**130**	PQAPAPAPAPAMVSA	2,756
**131**	PAPAPAPAMVSALAQ	2,777
**132**	APAPAMVSALAQAPA	4,802
**133**	PAMVSALAQAPAPVP	12,659
**134**	VSALAQAPAPVPVLA	5,824
**135**	LAQAPAPVPVLAPGP	2,657
**136**	APAPVPVLAPGPPQA	2,539
**137**	PVPVLAPGPPQAVAP	2,182
**138**	VLAPGPPQAVAPPAP	2,866
**139**	PGPPQAVAPPAPKPT	3,016
**140**	PQAVAPPAPKPTQAG	2,671
**141**	VAPPAPKPTQAGEGT	1,559

**142**	PAPKPTQAGEGTLSE	916
**143**	KPTQAGEGTLSEALL	1,205
**144**	QAGEGTLSEALLQLQ	1,177
**145**	EGTLSEALLQLQFDD	676
**146**	LSEALLQLQFDDEDL	492
**147**	ALLQLQFDDEDLGAL	384
**148**	QLQFDDEDLGALLGN	965
**149**	FDDEDLGALLGNSTD	801
**150**	EDLGALLGNSTDPAV	1,449
**151**	GALLGNSTDPAVFTD	1,042
**152**	LGNSTDPAVFTDLAS	614
**153**	STDPAVFTDLASVDN	1,054
**154**	PAVFTDLASVDNSEF	1,019
**155**	FTDLASVDNSEFQQL	1,031
**156**	LASVDNSEFQQLLNQ	1,528
**157**	VDNSEFQQLLNQGIP	1,097
**158**	SEFQQLLNQGIPVAP	2,355
**159**	QQLLNQGIPVAPHTT	1,052
**160**	LNQGIPVAPHTTEPM	841
**161**	GIPVAPHTTEPMLME	696
**162**	VAPHTTEPMLMEYPE	317
**163**	HTTEPMLMEYPEAIT	589
**164**	EPMLMEYPEAITRLV	952
**165**	LMEYPEAITRLVTGA	16,075
**166**	YPEAITRLVTGAQRP	31,436
**167**	AITRLVTGAQRPPDP	1507
**168**	RLVTGAQRPPDPAPA	20,988
**169**	TGAQRPPDPAPAPLG	641
**170**	QRPPDPAPAPLGAPG	1,592
**171**	PDPAPAPLGAPGLPN	1,789
**172**	APAPLGAPGLPNGLL	2,138
**173**	PLGAPGLPNGLLSGD	9,475
**174**	APGLPNGLLSGDEDF	1,127
**175**	LPNGLLSGDEDFSSI	908
**176**	GLLSGDEDFSSIADM	972
**177**	SGDEDFSSIADMDFS	1,095
**178**	EDFSSIADMDFSALL	1,079
**179**	SSIADMDFSALLSQI	3,301

Footnote S1: The signal intensities were measured using the macro "Protein Array Analyzer" from Image J. The measured values ranged between 174,660 and 1,368 and the threshold was set at 100,000 to determine the "binders" (highlighted in bold).

Table S2: Sequences of the length analysis of the peptide GTVRISLVTKDPPHRPHP (spot 24-25 of Table S1) with the corresponding signal intensities.

Spot	Sequences	Signal intensities
**1**	GTVRISLVTKDPPHRPHP	**163,329**
**2**	GTVRISLVTKDPPHRPHP	**155,341**
**3**	TVRISLVTKDPPHRPHP	**176,564**
**4**	GTVRISLVTKDPPHRPH	97,625
**5**	VRISLVTKDPPHRPHP	**154,344**
**6**	GTVRISLVTKDPPHRP	12,406
**7**	RISLVTKDPPHRPHP	80,358
**8**	GTVRISLVTKDPPHR	204,526
**9**	ISLVTKDPPHRPHP	13,386
**10**	GTVRISLVTKDPPH	7,567
**11**	SLVTKDPPHRPHP	6,839
**12**	GTVRISLVTKDPP	43,939
**13**	LVTKDPPHRPHP	6,025
**14**	GTVRISLVTKDP	**205,800**
**15**	VTKDPPHRPHP	6,681
**16**	GTVRISLVTKD	**194,346**
**17**	TKDPPHRPHP	11,797
**18**	GTVRISLVTK	**207,007**
**19**	KDPPHRPHP	4,225
**20**	GTVRISLVT	**116,165**
**21**	DPPHRPHP	5,033
**22**	GTVRISLV	86,789
**23**	PPHRPHP	33,601
**24**	GTVRISL	**192,825**
**25**	PHRPHP	43,456
**26**	GTVRIS	**182,383**

Footnote S2: The signal intensities were measured using the macro "Protein Array Analyzer" from Image J. The measured values ranged between 204,526 and 4,225 and the threshold was set at 100,000 to determine the "binders" (highlighted in bold).

Table S3: Sequences of the substitutional analysis of the peptide RISLVTKDPPHR (spot 8 of Table S2) with the corresponding signal intensities.

	wt	A	C	D	E	F	G	H	I	K	L	M	N	P	Q	R	S	T	V	W	Y
**R**	**235,694**	**238,793**	**127,596**	**81,357**	**143,476**	**252,566**	**218,304**	**210,702**	**245,336**	**216,342**	**194,232**	**128,334**	**171,537**	**157,859**	**126,219**	**154,406**	**122,333**	**158,344**	**90,064**	**110,324**	**184,267**
**I**	**242,524**	46,685	27,896	17,046	26,174	**242,951**	25,275	23,550	**204,314**	43,385	17,748	16,044	19,731	15,360	10,936	13,842	10,478	16,444	15,559	68,526	**129,418**
**S**	**235,716**	**123,843**	**123,227**	71,100	**89,021**	**209,297**	**111,795**	**187,921**	78,403	**220,393**	**142,432**	70,405	**93,854**	78,483	72,114	**105,376**	**102,245**	**86,615**	38,457	**172,503**	**150,337**
**L**	**243,425**	28,959	44,334	11,339	26,688	260,094	20,286	31,299	**179,375**	**110,969**	**226,036**	9,736	17,562	8,541	12,514	**209,074**	15,296	13,114	15,206	60,065	**135,667**
**V**	**244,267**	**134,552**	**172,798**	10,142	13,000	**259,269**	**125,566**	**179,689**	**248,441**	**254,221**	**250,285**	36,639	63,527	16,086	53,417	**258,955**	**139,077**	**82,823**	78,421	**96,252**	**181,625**
**T**	**254,778**	**205,268**	**191,761**	11,308	10,639	**258,321**	**91,170**	**106,881**	**182,493**	**223,221**	**260,069**	72,480	72,196	**91,405**	**109,130**	**258,088**	**200,616**	**135,333**	**159,581**	**163,355**	**244,860**
**K**	**240,018**	38,075	22,201	9,066	10,797	**157,916**	22,005	33,255	49,542	**82,786**	60,762	10,835	17,957	9,634	12,576	**220,024**	33,964	19,729	33,258	62,804	**81,459**
**D**	**257,412**	**256,119**	**248,841**	**190,846**	**194,358**	**259,774**	**256,060**	**242,065**	**245,766**	**253,495**	**239,364**	**159,748**	**183,109**	**226,288**	**233,417**	**259,525**	**254,240**	**245,975**	**248,769**	**207,670**	**254,179**
**P**	**234,015**	**228,093**	**121,136**	38,385	29,194	**252,090**	**131,070**	**123,348**	**101,715**	**199,670**	**135,333**	47,964	**179,302**	**80,982**	**129,163**	**233,743**	**108,972**	75,663	**84,584**	**158,776**	**210,525**
**P**	**219,612**	**242,816**	**205,163**	36,369	33,192	**243,331**	**185,362**	**144,490**	**152,488**	**182,055**	**147,196**	63,585	**163,300**	**91,793**	**87,004**	**227,875**	**138,821**	**122,769**	**84,888**	**124,651**	**180,037**
**H**	**223,624**	**208,702**	**105,009**	23,814	23,697	**199,377**	**143,658**	**136,995**	**111,858**	**192,353**	**133,839**	61,440	**110,257**	63,207	56,630	**186,130**	**105,542**	**98,069**	**91,735**	**123,222**	**149,297**
**R**	**226,039**	19,320	7,395	4,734	4,433	39,435	9,010	6,676	8,293	56,485	11,461	6,409	7,060	6,272	7,271	**101,766**	12,050	11,200	8,708	11,783	33,087

Footnote S3: The signal intensities were measured using the macro "Protein Array Analyzer" from Image J. The measured values ranged between 260,094 and 4,433, and the threshold was set at 80,000 to determine the "binders" (highlighted in bold). In yellow are highlighted the key amino acid positions of the peptide RISLVTKDPPHR tolerating only several replacements (not more than 5).
